# A novel dual-energy cone beam computed tomography device for assessment of jaw bone density

**DOI:** 10.1007/s11282-026-00928-4

**Published:** 2026-05-04

**Authors:** Vidhya Venkateswaran, Ritu Tiwari, Tara Aghaloo, Reuben Kim, Olga Bezouglaia, Sanjay M. Mallya

**Affiliations:** 1 Section of Oral and Maxillofacial Radiology, School of Dentistry, University of California Los Angeles, Los Angeles, CA USA; 2 Present Address: National Institute of Dental and Craniofacial Research, National Institutes of Health, Bethesda, MD USA; 3 Present Address: School of Dentistry, University of Texas Health Science Center at Houston, Houston, TX USA; 4 Section of Oral and Maxillofacial Surgery, School of Dentistry, University of California Los Angeles, Los Angeles, CA USA; 5 Section of Restorative Dentistry, School of Dentistry, University of California Los Angeles, Los Angeles, CA USA

**Keywords:** Dual-energy computed tomography, Spectral computed tomography, Cone beam computed tomography, Jaw bone density, Spectral cone beam computed tomography, Dual-energy cone beam computed tomography

## Abstract

**Objectives:**

Cone beam computed tomography (CBCT) provides morphological details of the dental hard tissues and bone. However, the relationship between object density and CBCT grey values is unreliable and limits application of CBCT for quantitative evaluation of bone density. This single center, open-label clinical trial investigated the validity of novel dual-energy CBCT device (DE-CBCT) to assess jaw bone density.

**Methods:**

The study analyses comprised 24 subjects who were examined with DE-CBCT and multidetector CT (MDCT)—an established standard for bone density assessment. MDCT and DE-CBCT scans of each subject were analyzed in the vendor’s software application for assessment of bone density at 10 discrete sites in the jaws. Two independent assessments were made.

**Results:**

The mean bone density assessed by DE-CBCT and MDCT were not significantly different (0.46 ± 0.36 g/cc versus 0.48 ± 0.31 g/cc, respectively). Bone density assessments from DE-CBCT correlated well with MDCT assessments (Pearson’s correlation *r* = 0.92, *p* < 0.0001). Bland-Altman analyses demonstrated higher agreement between the two techniques at densities great than 0.6 g/cm^3^. However, at lower densities the limits of agreement were broad.

**Conclusions:**

DE-CBCT is a promising approach to assess bone density in the jaw bones. The investigational device performs best in well-trabeculated and cortical bone, with reduced reliability in sparsely trabeculated regions. Future refinements in this technology will be needed to expand the range of reliability for reliable assessment of bone density.

**Clinical trials registration:**

The study is registered in clinical trials.gov, NCT04686084.

## Introduction

Computed tomography (CT) is an x-ray-based imaging technique that produces sectional images from x-ray attenuation data. In all CT techniques, a collimated x-ray source and a detector revolve around the patient, registering x-ray attenuation information at several hundred angles. Complex mathematical algorithms translate this attenuation data into a three-dimensional (3D) map that spatially locates the attenuating structures [1, 2]. Each voxel in the CT dataset is assigned a CT number based on its average linear attenuation coefficient. CT numbers are expressed in Hounsfield units (HU), a dimensionless unit represented on a scale with fixed CT numbers assigned to air (−1000) and water (0), respectively [1, 2]. The upper end of the HU scale varies with image bit-depth. A standard MDCT 12-bit image will depict 4096 grey shades, mapped on the HU scale from − 1024 to 3071.

The CT number of a voxel depends on its component tissue (effective atomic number and physical density), the x-ray energy spectrum, and the reconstruction algorithm used. Thus, except for air and water, the CT numbers of tissues and materials will vary depending on scanner settings and manufacturer [3, 4]. Clinically, applications of HU measurements include evaluation of adipose content in fatty liver degeneration, evaluation of lung nodules and estimation of bone mineral density. However, practical use of HU is limited by its reliability—an object’s radiopacity varies across scanners, imaging protocols and image reconstruction kernels used. Indeed, specialized CT techniques must be used for evaluation of osteopenia and osteoporosis, which require high fidelity of the HU scale in the region of bone attenuation. Such techniques require special phantoms and analytical software.

Dual-energy CT (DECT) is a contemporary CT technology that directly addresses limitations of single spectrum MDCT imaging. It capitalizes on a fundamental physical principle of x-ray attenuation—high-atomic number tissues (e.g., cortical bone) display marked energy-dependent attenuation differences, whereas soft tissue attenuation varies less with energy. DECT exploits this differential attenuation by acquiring and analyzing attenuation data from two energy spectra to infer material composition from paired low- and high-energy attenuation measurements [5, 6]. This translates into better estimates of attenuation coefficient, potentially allowing distinction between bone with different calcium contents to derive local bone density.

Cone beam CT (CBCT) is widely used in dentistry for a variety of diagnostic tasks [7]. Relative to MDCT, CBCT contrast resolution is poor—an inherent limitation of the wide beam geometry and scattered photons striking the detector [7]. Several studies have demonstrated that HU/grey values from CBCT volumes are unreliable and vary with scanner settings and anatomic location, limiting quantitative density assessment across an imaged volume [8–14]. For example, the CT number of the same object differs depending on its anatomical location [8, 14]. Objects with low x-ray attenuation exhibit greater variability [8, 14], and grey values are markedly influenced by scanner settings [10]. To this end, dual-energy or spectral imaging could mitigate these limitations by sampling attenuation at two energies within the same scan and leveraging material-specific attenuation differences.

A recent clinical trial suggests that dual-energy CBCT (DE-CBCT) can yield quantitative bone measurements that agree with multidetector CT (MDCT). In this pilot study of 15 adults, jawbone density measured with a newly developed DE-CBCT (RCT720, Ray Co. Ltd.) showed an excellent correlation (*r* = 0.982, *p* < 0.001) with calibrated MDCT, with no significant difference in measured bone density (mean difference = 0.04 ± 0.02 g/cm³) [15].

Beyond estimating bone density, spectral CT imaging may reduce artifacts around metal. Zhu et al. described a newly developed spectral CBCT scanner (UEG Realtime; UEG Imaging Co. Ltd., Shanghai, China) that uses alternating spectra (80kVp/110 kVp) with dynamic copper filtration [16]. They found that the device reduces metal artifacts and improves contrast-to-noise ratio around metallic restorations, especially for smaller metallic objects. However, the trade-off was increased noise in regions without metal [16].

The present study uses an investigation DE-CBCT device (RCT720, Ray Co. Ltd.) with rapid kVp switching (80kVp/90kVp) to provide paired-spectrum data for density estimation after hydroxyapatite-based calibration. By validating DE-CBCT measurements against MDCT at multiple jaw sites, we sought to determine whether this device can deliver clinically useful, quantitative bone density assessments suitable for applications such as implant treatment planning and monitoring osseous healing.

## Materials and methods

### Subjects

This clinical trial was conducted at the UCLA School of Dentistry and enrolled 26 subjects (14 male and 12 female), aged 21 to 81 years (Table 1). The inclusion criteria were: (1) Patients who need CT imaging for dental diagnosis and/or treatment planning, (2) Males or females aged older than 21 years, (3) Can follow instructions to be positioned into the CT scanner, (4) Can remain physically immobile during the CT scan acquisition, and (5) Voluntarily sign and date the informed consent. The exclusion criteria were: (1) Pregnancy, (2) Patients who are unable to comprehend the risks of the study to provide informed consent, and (3) Extensive dental restorations, maxillofacial prosthesis, or orthopedic hardware that likely may cause artifacts and degrade quality, as determined by the study radiologists.

The study was approved by the UCLA Institutional Review Board. The study is registered in clinical trials.gov (NCT04686084, Unique Protocol ID: 20-001768).

**Table 1:** Age and gender demographics of study subjects

Age (years)	Female			Male		
	Recruited	Completed	Excluded	Recruited	Completed	Excluded
≤ 45	4	4	-	5	4	-
> 45 to ≤ 65	4	4	-	5	5	1
> 65	4	4	-	4	4	-
TOTAL	12	12	-	14	13	1

## CT scanners and protocols

Each study subject was scanned with a standard of care MDCT scan and the investigational DE-CBCT. MDCT scans were acquired on a Siemens SOMATOM Definition AS 64-slice CT with the following exposure factors: 120kVp, 150mAs, 0.5s; and reconstructed at a slice thickness of 0.6 mm with the HR56 reconstruction bone kernel. DE-CBCT scans were acquired using the investigational CBCT device (RCT 720, Ray Co. Ltd, Seoul, S. Korea) using a 10 cm x 10 cm field of view, a dual kVp setting (80 kV and 90 kV) and 17 mA. Scans were reconstructed at 0.3 mm slice thickness as per the manufacturer’s process. Volumetric data from the DE-CBCT and MDCT scans were exported in DICOM format and used for subsequent bone density analyses as described below.

To calibrate CT numbers and bone density, we used a commercially available phantom (QRM, Moehrendorf, Germany). This cylindrical polymethacrylate phantom is 70 mm in diameter and 70 mm in height and simulates soft tissue attenuation within the head. Three hydroxyapatite (HA) inserts (16 mm d x 7 mm h) with concentrations of 0.803, 0.997 and 1.168 g/cm^3^ were placed in the phantom. The phantom was scanned asynchronously in the MDCT and DE-CBCT devices using the same exposure factors and reconstruction parameters used for subject exposures following standard practice parameters [17]. Phantoms scans were done immediately prior to the subject scan.

### Bone density assessment

**Fig. 1:**
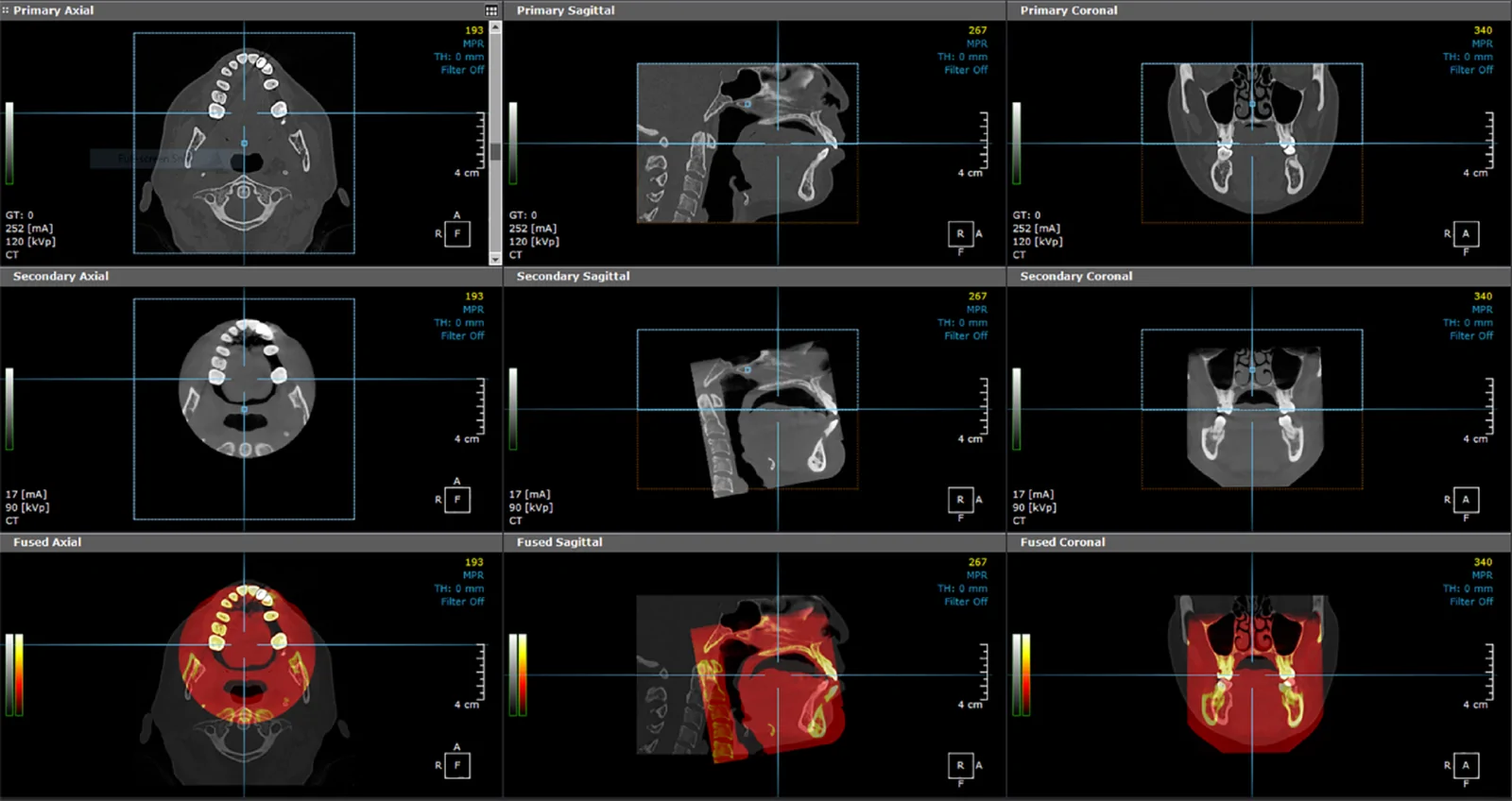
SMDBDA software provided with the investigational device. Orthogonal sections from MDCT (labelled as primary) and DE-CBCT (labelled as secondary) data are displayed. Superimposition of the two volumes is displayed in the bottom panel. The shaded area identifies the region of overlap between the MDCT and DE-CBCT scans. The maxilla and cranial base are superimposed to prepare for bone density assessments in the maxilla

**Fig. 2:**
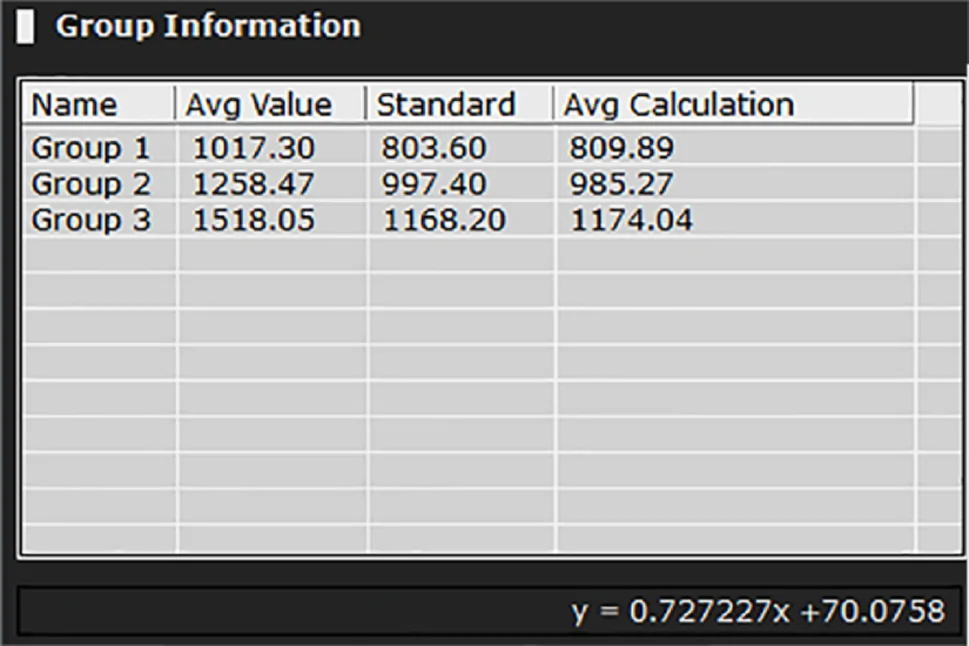
Calibration of CT numbers to standard concentrations of hydroxyapatite. The software provides a calibration curve using the three hydroxyapatite concentrations in the phantom. Separate calibration curves are done for each MDCT and DE-CBCT phantom acquisition and applied to measurements on individual subject scans

Volumetric data from the DE-CBCT and MDCT scans were exported in DICOM format and imported in SMDBDA software provided with the investigational device. Modules within the software allow for accurate registration and superimposition of the MDCT and DE-CBCT scans. The accuracy of the superimposition was examined by close visual examination of the superimposed area and refined with a combination of repeated manual and automatic registration until the anatomic structures in the region were superimposed with no discordance in anatomic outlines. The maxilla and mandible were superimposed separately (Fig. 1). Modules within the software were used to measure the CT numbers in the HA cylinders of the phantom and used and compute a calibration curve that was applied individually to the MDCT and CBCT scans (Fig. 2).

**Fig. 3:**
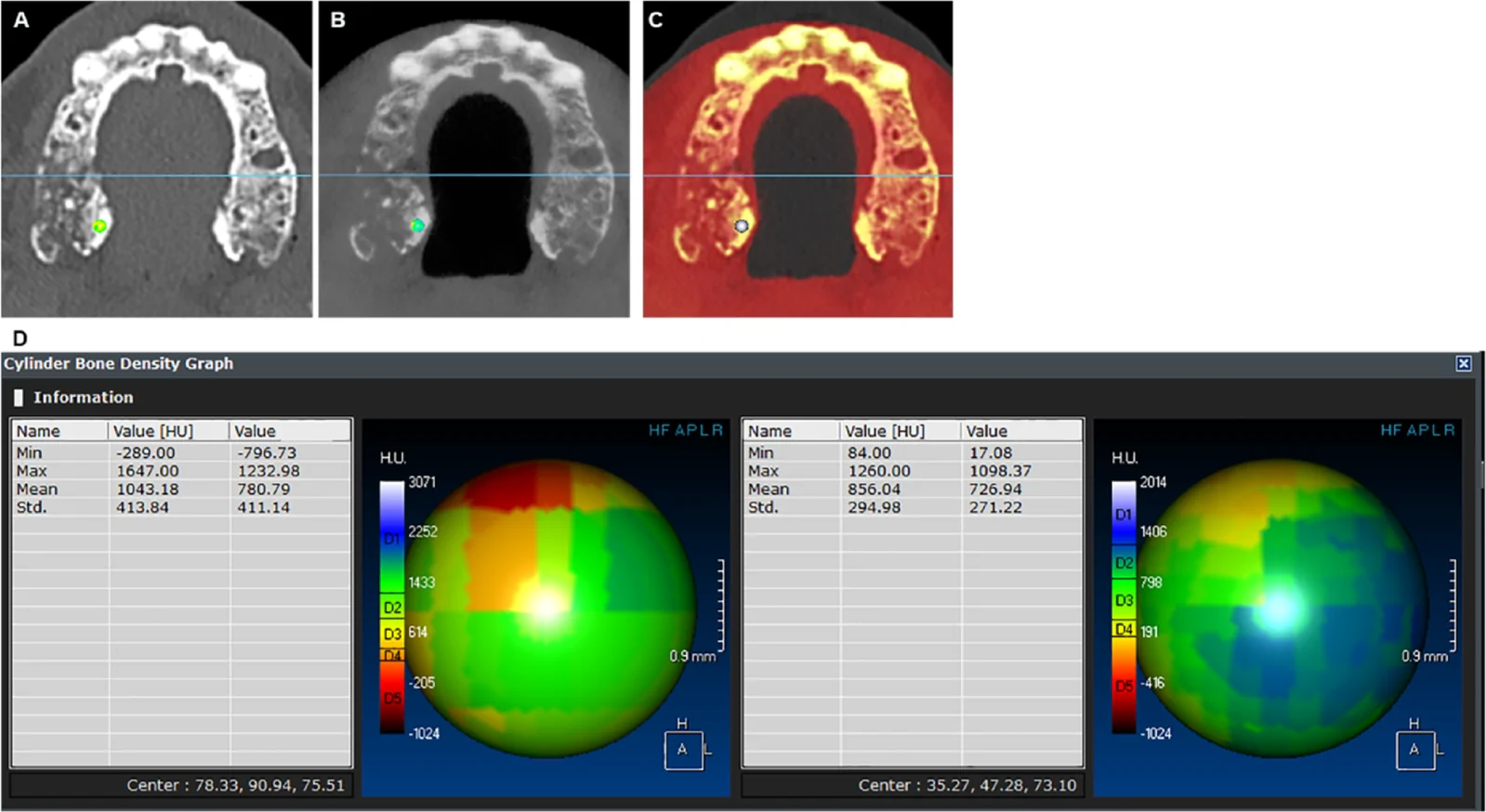
Bone density assessment. **A** MDCT. **B** DE-CBCT. **C** Superimposition of the MDCT and DE-CBCT scans. Note the region of interest in the cortical bone of the right maxillary tuberosity region. **D** Presentation of bone density assessments. The calculated bone density for the MDCT scan (left) and DE-CBCT scan (right) are displayed. Graphical representations of the HU values represented in the spherical region of interest are also displayed. The mean values (780.79 mg/cm^3^ and 726.94 mg/cm^3^) were recorded

Subject MDCT and DE-CBCT scans were imported into the software. Following superimposition, the user directed the software to apply the specific calibration files for the MDCT and DE-CBCT scans. Using tools with the software, the investigator selected a spherical region of interest (ROI, 3–5 mm diameter) within the jaw bone. This ROI was placed at locations in the maxilla and mandible to encompass regions of cortical and/or trabecular bone. Investigators visually examined the region to confirm absence of artifacts from restorations. Investigators visually examined the superimposition of CT images in the ROI to confirm that the measurements were made at identical locations on the MDCT and the DE-CBCT scans. Software tools provided calculated bone density for the ROI in each scan, based on the individual calibration algorithms created for the MDCT and DE-CBCT scans. Paired measurements and the location of the ROI in the jaw were recorded (Fig. 3).

### Statistical analyses

Data were analyzed using the statistical package in Prism9 (Graphpad, San Diego, CA). For analysis of agreement between the two methods of bone density assessment, Bland Altman analyses were used [18]. 

## Results

### Subjects

The study recruited 26 patients, 14 male and 12 female, aged 21 to 85 years. One subject was lost to follow-up and did not receive the investigational CT scan. One subject was excluded because of artifacts on the CT scan that compromised analyses. The eventual study analyses included 24 subjects, 12 male and 12 female (Table 1). The mean age was 54.2 ± 20 years mean ± standard deviation). Mean ages of female and male subjects were not statistically different (53.8 ± 21 years vs. 54.5 ± 20.1 years, respectively, *p* > 0.05). There were no adverse events associated with the DE-CBCT and MDCT scans.

### Bone density analysis

Paired bone density assessments were made by independently by 2 investigators at 10 discrete locations within the jaws of each subject. Care was taken to exclude areas with imaging artifacts. In total, 480 paired assessments were made. The bone density assessments spanned the full scale of bone densities to a maximum of 1.5 g/cm^3^ (as assessed on MDCT scans).

**Fig. 4:**
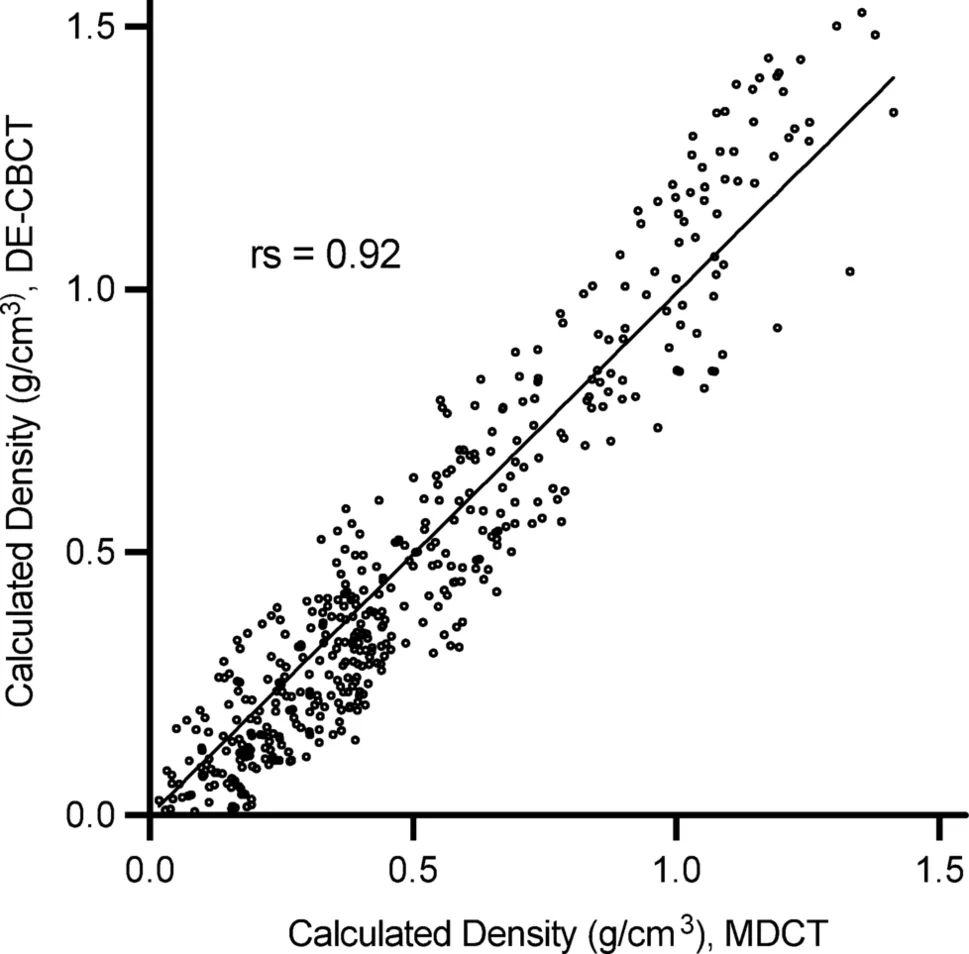
Correlation between MDCT and DE-CBCT derived bone density assessments

Table 2 provides summary statistics of the bone density assessments from the MDCT and DE-CBCT scans. The means of bone density assessed by DE-CBCT (0.46 ± 0.36 g/cm^3^) and by MDCT (0.48 ± 0.31 g/cm^3^) were not significantly different (*p* > 0.05). Overall, there was strong linear correlation between bone density assessments provided by MDCT and by DE-CBCT (Fig. 4, Spearman’s correlation *rs* = 0.92, *p* < 0.0001).

**Table 2:** Descriptive statistics of all bone density assessments (g/cm^3^) made on MDCT and CBCT scans

	ASSESSMENT #1		ASSESSMENT #2	
	DE-CBCT	MDCT	DE-CBCT	MDCT
Mean ± SD	0.45 ± 0.36	0.47 ± 0.32	0.48 ± 0.36	0.49 ± 0.31
Median	0.33	0.39	0.36	0.40
Range	0.01–1.49	0.02–1.36	0.01–1.53	0.03–1.41
25th percentile	0.19	0.22	0.20	0.27
75th percentile	0.61	0.67	0.68	0.65

**Fig. 5:**
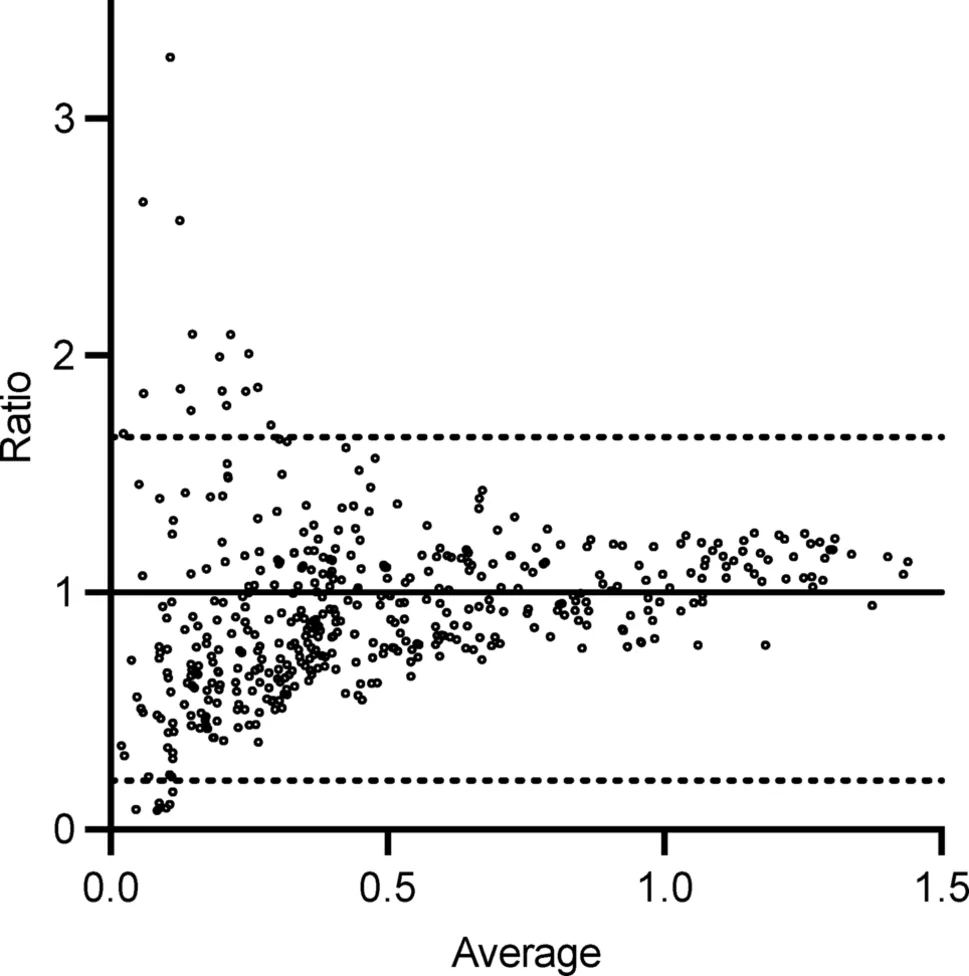
Bland Altman analysis of all bone density assessments. The Y-axis plots the ratio of the MDCT and DE-CBCT derived bone density assessments. The X-axis plots the average of the MDCT and DE-CBCT assessments. The solid line indicates a ratio of 1 and 95% limits of agreement (dotted lines) are indicated. Note that data at higher densities are clustered closer to 1

We next examined the overall agreement between bone density assessed by DE-CBCT and MDCT using Bland-Altman plots. In this analysis, the ratio of the bone density assessments by the two CT technologies are plotted on the Y axis and the averages of the paired measurements are plotted on the X-axis. A ratio close to 1 indicates better agreement between the DE-CBCT and MDCT bone density assessments. The mean ratio of the bone density assessed by DE-CBCT and MDCT was 0.93 ± 0.37, demonstrating an overall high agreement. The range of 95% limits of agreement was broad and ranged from 0.2 to 1.7 (Fig. 5). More than 95% of the data point lie between the limits of agreement. There was a notable trend of ratios closer to 1with higher bone density assessments (Fig. 5), indicating strong agreement at higher bone density.

**Fig. 6:**
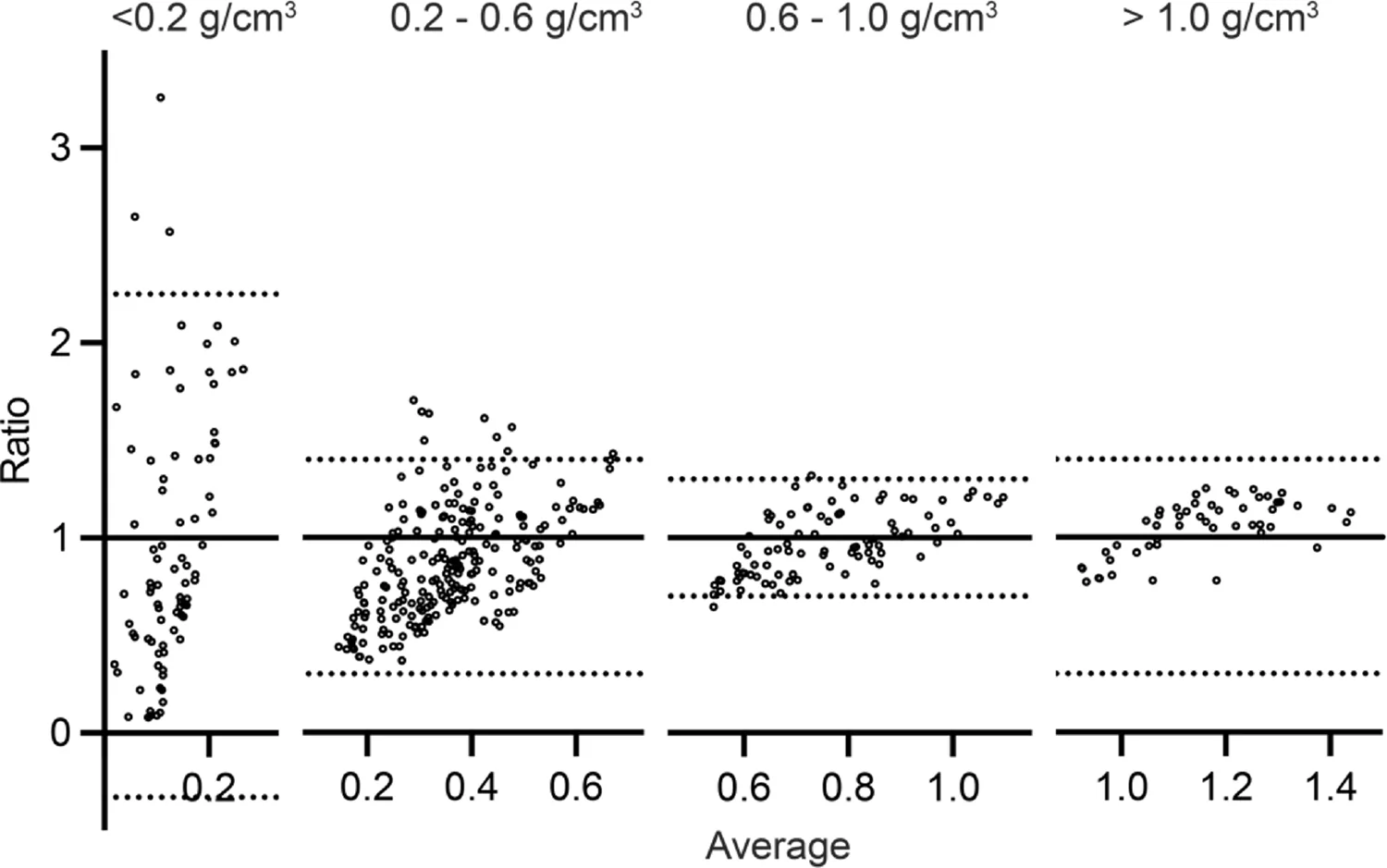
Bland Altman analysis of bone density assessments by category. The Y-axis plots the ratio of the MDCT and DE-CBCT derived bone density assessments. The X-axis plots the average of the MDCT and DE-CBCT assessments. The solid line indicates a ratio of 1 and 95% limits of agreement (dotted lines) are indicated. Note that data at densities higher than 0.6 g/cm^3^ are clustered closer to 1 with narrow limits of agreement

To further examine performance of DE-CBCT at different bone densities, we categorized bone density into four groups based on the MDCT-derived bone density assessments. Table 3 shows the results of the Bland Altman analyses for each of these density groups. For the lowest density group (bone density < 0.2 g/cm^3^), the variation between MDCT and DE-CBCT assessments was highest. At density ranges of more than 0.2 g/cm^3^, there was better agreement between the MDCT and DE-CBCT assessments of bone density. Notably, the limits of agreement were narrowest at bone densities of 0.6 g/cm^3^ and higher (Fig. 6).

**Table 3:** Bland Altman analyses at different ranges of bone densities

HA density range, g/cm^3^	Mean ratio ± SD	95% Limits of agreement
0–1.5	0.93 ± 0.37	0.2–1.66
< 0.2	0.96 ± 0.66	−0.33 to 2.25
0.2 to < 0.6	0.87 ± 0.29	0.3–1.4
0.6 to 1.0	0.98 ± 0.17	0.7–1.3
> 1.0	0.98 ± 0.2	0.3–1.4

## Conclusion

This study investigated the validity of a novel dual-energy cone beam computed tomography device (DE-CBCT) to assess jawbone density. In this clinical trial, all subjects successfully completed the study with no adverse events.

Our study used MDCT as the reference standard for bone density assessment. Despite the energy dependence of CT numbers, MDCT imaging with a single energy spectrum is used to assess bone density at skeletal sites. In these quantitative CT examinations, a phantom object with relevant concentrations of hydroxyapatite is scanned, either synchronously with the patient, or asynchronously, as an independent scan. Both phantom scanning approaches have been validated and practice parameters for quantitative CT have been developed [17].

Bone densities derived from DE-CBCT were strongly correlated with MDCT values (Fig. 1; Pearson *r* = 0.92, *p* < 0.0001). Group means did not differ significantly (DE-CBCT 0.46 ± 0.36 g/cm³ vs. MDCT 0.48 ± 0.31 g/cm³), indicating that, on average, DE-CBCT provided density estimates comparable to calibrated MDCT [8, 9, 13, 14, 19].

However, a strong correlation does not necessarily imply agreement and interchangeability between the two techniques. Thus, we used Bland Altman analyses to examine the agreement between the two measurement techniques. The overall mean ratio was 0.93 ± 0.37 (Fig. 5), suggesting acceptable but variable agreement; ratios < 1 indicate a modest tendency for DE-CBCT to yield slightly higher densities than MDCT on average. Agreement was not uniform across the measurement range. At lower densities, individual ratios were more dispersed and deviated from 1.0, whereas at ≥ 0.6 g/cm³ the data clustered tightly around unity with narrower limits of agreement (Fig. 6). This pattern implies that DE-CBCT performs best in well-trabeculated and cortical bone, with reduced reliability in sparsely trabeculated regions. This limits potential clinical application of DE-CBCT, for example, to quantitatively assess osseous healing, where measured bone densities are low.

Potential applications for such assessments include implant treatment planning, evaluation of bone demineralization and mineralization, and post-surgical healing. Although current practices do not rely on quantitative estimates of bone density, application of this technique to the stated diagnostic tasks may help identify situations that would benefit from added bone density assessments. Notably, agreement between MDCT and DE-CBCT was low when assessing bone densities lower than 0.2 g/cm^3^ (Fig. 6). These densities represent measurements made at areas of sparse trabecular bone and were evident on radiological evaluation. Such areas of trabecular sparseness and demineralization are usually apparent on visual examination of CT scans and added value from bone density measurements may not be immediately applicable for patient management.

The narrow spectral separation (80 kVp vs. 90 kVp) likely contributed to the range-dependent behaviour. The RCT720 DE-CBCT device uses rapid switching of beam energy between 80 kVp and 90 kVp, representing mean effective energies of approximately 27 keV and 30 keV. This energy difference may be insufficient to robustly disentangle photoelectric and Compton components, particularly in low-density bone where the signal-to-noise is lower. In contrast, our MDCT reference was acquired at 140 kVp, and the different spectrum, filtration, and reconstruction kernels—despite calibration—could accentuate inter-modality differences at the low end of the attenuation range.

A second limitation of this study is the experimental approach to superimpose the MDCT and DE-CBCT scans. To ensure that measurements were made at the precise same location on the MDCT scan and the DE-CBCT scan, we used custom software for image registration and superimposition. Although all registrations were curated, small undetected mis-registrations remain possible. This is salient for spherical ROIs of 3–5 mm: in sparse trabeculation, sub-millimeter shifts can alter the fraction of cortical struts or marrow spaces included, perturbing measured density and widening the limits of agreement. Additionally, differences in voxel size, point-spread function, and partial-volume effects between scanners could differentially bias low-density measurements.

In summary, this study demonstrates that bone density inferred from DE-CBCT scans show strong agreement at densities ≥ 0.6 g/cm³ and highlights the need for technical refinements to improve performance in low-density trabecular bone. With technical improvements, DE-CBCT could be applied for quantitative applications in mineralized regions of the jaws.

## Data Availability

The clinical trial is registered at ClinicalTrials.gov ID NCT04686084.
